# Perceptions of Illicit Tobacco Sources Following a Proposed Reduction in Tobacco Availability: A Qualitative Analysis of New Zealanders Who Smoke

**DOI:** 10.1093/ntr/ntad034

**Published:** 2023-03-04

**Authors:** Janet Hoek, Anna Graham-DeMello, Nick Wilson

**Affiliations:** Department of Public Health, University of Otago Wellington, Wellington, New Zealand; Department of Preventive and Social Medicine, University of Otago Dunedin, Dunedin, New Zealand; Department of Public Health, University of Otago Wellington, Wellington, New Zealand

## Abstract

**Introduction:**

Tobacco companies claim that substantially reducing tobacco retail outlets in Aotearoa New Zealand will increase illicit tobacco trade and crime. However, we know little about whether people who smoke anticipate using illicit tobacco once this measure is implemented. Exploring current illicit tobacco use and expected market development would clarify the likely scale of this potential problem.

**Aims and Methods:**

We undertook online in-depth interviews with 24 adults who smoke and explored their experiences of illicit tobacco, perceptions of illicit market growth once legal tobacco became less available, intentions to engage in this market, and potential measures that could curb illicit market development. We interpreted the data using a qualitative descriptive approach.

**Results:**

Few participants had purchased illegally imported or stolen tobacco. While most did not know how to access illicit tobacco products, many expected illicit trade and crime would increase, if legal tobacco became difficult to access. While cheaper tobacco appealed to many, most perceived illicit supply routes as unsafe and saw products obtained via these sources as likely to be of poor quality. Few suggested measures to control illicit markets, though a minority called for social reforms to reduce poverty, which they thought fueled illegal practices.

**Conclusions:**

Although illicit trade may appear to threaten new policy initiatives, participants’ limited knowledge of these markets and concerns regarding product safety suggest illegal tobacco may pose less of a threat than tobacco companies have claimed. Policy makers should not be deterred from reducing tobacco availability by industry arguments.

**Implications:**

Although participants believed illicit trade would increase if the number of tobacco retailers was substantially reduced, few anticipated purchasing illegal tobacco. They viewed supply routes as unsafe and product quality as likely to be low. Industry predictions that illicit tobacco trade will grow if tobacco becomes less available do not reflect how people who smoke expect to engage with these markets and should not deter the introduction of retail reduction measures.

## Introduction

Tobacco products’ wide availability has helped normalize smoking as an accepted social practice.^1^ However, as more countries consider tobacco endgame goals, attention has turned to address the historical anomaly that allows tobacco to be sold as a consumer product with few purchase restrictions.^2^

Reducing tobacco’s ubiquitous retail presence occurred first among municipalities; Beverley Hills and Manhattan Beach (US) passed ordinances ending the sale of tobacco products.^3,4^ Within California, San Francisco had earlier limited the number of tobacco retail permits granted, though allowed “grandfathering.”^5^

Nationally, only Hungary has thus far implemented legislation that substantially reduces the tobacco retail outlets,^6^ though other countries are considering supply reduction measures. For example, the Netherlands disallowed sale of tobacco products from vending machines in 2022, will stop online sales of tobacco products in 2023, end tobacco sales from supermarkets from 2024 and, from 2030 onwards, phase out tobacco sales from service stations and convenience stores.^7,8^ The Aotearoa New Zealand (NZ) Parliament (Aotearoa is the indigenous Māori people’s name for the country more widely known as New Zealand) has recently passed legislation allowing no more than 600 stores to sell tobacco, thus reducing the estimated 6000 to 8000 stores currently selling tobacco products by around 90%.^9^

### Illicit (Black Market) Trade: Rhetoric and Reality

Studies suggest limiting tobacco retailer numbers will reduce smoking prevalence,^10^ decrease youth uptake,^11,12^ and support cessation^13^; however, tobacco companies have consistently opposed these plans.^14^ Analyses of industry opposition have identified common discursive claims that exaggerate policies’ potential costs while dismissing or denying their potential benefits.^15^ As Ulucanlar et al. noted, this approach aims to create “a comprehensive and credible narrative of undesirability for the policy by generating tailored arguments covering many different social domains” (p.6).^15^ Among claims used to amplify perceived costs, tobacco companies have argued that supply reduction measures will stimulate illicit tobacco markets,^16^ lead to criminal activity, and stretch limited police resources.^15,17^

Illicit tobacco may come from varied sources, including products smuggled from overseas, resold stolen domestic tobacco, or on-sold homegrown tobacco. Regardless of source, illicit trade maintains tobacco availability, typically at a lower cost than legally sold tobacco products, and undermines supply reduction policies.^18^ Because the risk of unintended outcomes may deter policy development, critically evaluating tobacco companies’ arguments is crucial. Such analyses have found industry reports of illicit tobacco trade were low quality, and characterized by undeclared conflicts of interest,^19^ or methodological flaws likely to inflate the estimates reported.^16,20,21^ Yet despite these documented shortcomings,^19,22,23^ the illicit tobacco trade narrative often piques media interest and gains political traction.^24–27^

Within NZ, media reports of rising illicit trade do not align with independent studies. Using NZ Customs data on tobacco importation and seizures, one study examining the impact of annual excise tax increases on illicit tobacco trade estimated that illicit products constituted only 1.8% to 3.9% of total national tobacco consumption in NZ in 2013.^28^

NZ studies of discarded tobacco packaging also reported that foreign packs represent only a small proportion of all littered packs collected. A 2009 analysis found 42 foreign packs (3.2%) among the 1310 littered packs collected; this sample may have included packs legitimately imported and then discarded by tourists, thus potentially overestimating illicit tobacco use.^29^ A replication undertaken in 2012 to 2013 found 5.8% of the 1673 littered packs collected were foreign, though the authors noted this increase may reflect rising tourist numbers.^30^ More recent work, undertaken during COVID-19 travel restrictions when international tourism had stopped, estimated the national prevalence of foreign packs at 5.4%, though two major cities had higher concentrations of illicit packs.^31^

Differences between industry estimates and independent analyses of illicit trade may reflect an industry strategy to impede policy.^22,23^ Implying the illicit tobacco market is burgeoning may increase public anxiety about crime and reduce support for retail reduction policies. However, while independent studies suggest that smuggled illicit tobacco remains a relatively minor problem in NZ, rising “ram raids” that target small retailers may provide a source of illegal tobacco.^32^ Sales of homegrown tobacco have also reportedly increased and supported illicit market trade.^33^

These changes make it timely to explore knowledge of illicit tobacco trade and its future role, including anticipated involvement in the market when supply restrictions come into effect. We aimed to inform international supply reduction policies by exploring illicit trade within NZ.

## Methods

Data came from a larger qualitative study exploring NZ’s supply reduction policy among a sample of NZ adults who smoked.^34^ Daily smoking prevalence in NZ is eight percent^35^; reflecting sustained excise tax increases, plain packaging, and smoke-free areas, among other recent policies.^36^ Regulations limit homegrown tobacco production to 5kg annually and disallow sales, and alternative nicotine sources, via vaping products, are widely available.

### Sample

We recruited participants using social media and referrals; eligible participants were aged 18 or older and smoked at least five cigarettes a day. Interested people were directed to an online survey where they provided details of their age, gender, ethnicity, current tobacco consumption, location, and main tobacco purchase outlets (see Supplementary File 1). We used this information to structure the sample and maximize diversity.

We sent eligible participants a copy of an information sheet and consent form, then phoned them to assess their interest, answer any questions, establish a relationship and, if appropriate, arrange an online interview. We recruited participants primarily from Dunedin (a city in NZ’s South Island with a population of ~114 000) and Hamilton (a city on the North Island, with a population of ~178 000). We offered each participant a NZ$40 voucher to recognize their time and assistance.

### Interview Guide

Interviewers provided background information on themselves and offered to begin each interview with a karakia (a Māori prayer that creates a harmonious space for discussions). The interview explored participants’ smoking history and tobacco sources, and probed how they expected to respond to the proposed retail reduction policy (Supplementary File 2). We probed how participants anticipated they and others might locate and use illicit tobacco sources, their perceptions of the policy’s impact on illicit tobacco trade, and their views on possible responses to the challenges they foresaw.

A.D.M. and J.A.H. conducted interviews either online (via the Zoom e-conferencing platform) or by phone, depending on participants’ preferences. Interviews took place between April and June 2022 and lasted between 37 and 119 minutes. With participants’ permission, we recorded the interviews and used an online program (Otter.ai.) to transcribe these into anonymous verbatim records. A research assistant checked each transcript for accuracy. We wrote summary notes and analytic memos following each interview and included these in our analyses as we reflected on our roles as health researchers who support measures that will reduce smoking prevalence. Participants received a copy of their transcript and could edit these if they wished (none did). In total, we completed 24 interviews; recruitment ended when two consecutive interviews had not elicited new ideas. A departmental ethics reviewer approved the study as a low-risk project on behalf of the University of Otago Human Ethics committee (reference D22/050); all participants gave oral consent.

### Data Analysis

A.D.M. led initial coding with J.A.H., who later identified and re-coded material probing illicit tobacco into more detailed categories, which she reviewed with A.D.M.. We interpreted the data using qualitative description; this inductive approach remains close to the data and suited our goal of providing a thorough overview of our findings.^37^ We used NVivo release 1.6.2 to manage the initial coding then transported material to Microsoft Word to develop codebooks. We assigned each participant a pseudonym.

## Results

We outline participants’ characteristics in Table 1 and then present and illustrate four themes: increasing marginalization; complex supply networks, limited knowledge and experience, and formulating specific and societal solutions. We provide a detailed codebook in Supplementary File 3.

**Table 1.** Participant Characteristics

Characteristic	Number
Age group (y)	
18–34	12
35–50	8
>50	4
Gender (total sample)	
Female	15 (7 Māori, [Indigenous])
Male	8 (4 Māori)
Gender diverse	1
Ethnicities (identification with multiple ethnicities possible)	
Māori	11
New Zealand European	16
Pacific	1
Current tobacco consumption	
<10 cigarettes per day	7
≥10 cigarettes per day	17
Place of residence	
Dunedin is within 10 km radius of central location	10
Dunedin outside 10 km radius of central location	4
Hamilton is within 10 km radius of central location	9
Hamilton outside 10 km radius of central location	1

### Increasing Marginalization

Several participants thought illicit tobacco trade would grow as tobacco became less available and people unable to quit smoking sought tobacco from other sources. They expected illicit sources to replace outlets that formerly sold tobacco, and anticipated rising crime and gang power as people entered a new world of illegal practices. Others feared people driven by desperation would steal tobacco, placing at risk those who smoked and outlets authorized to sell tobacco.

Leanne explained how the policy would move people across the boundary of lawfulness: “You are giving people a choice between breaking the law and feeding their addiction. And the group that we’re concerned about will feed their addiction so you’re teaching them to break the law. So, you move a whole sector of society, who currently is simply an addict, into becoming a criminal addict…[and]… once you’ve stepped across into one criminal activity, there’s nothing to stop you [engaging in more criminal activity].” Yet, while she anticipated others would become embroiled in illicit supply networks, Leanne differentiated between these people and herself: “the fact that I’m an addict doesn’t actually put me into the seedy side of doing deals on street corners… I am not the kind of person to actually associate with people hocking things off on street corners.”

Many participants shared Leanne’s concerns that accessing tobacco would become a criminal activity; they anticipated the police would be “arresting people for buying a pouch of tobacco…[which would be] a whole waste of money and resources” (Atarangi). These participants thought police would struggle to manage illicit tobacco supply routes; Elsie explained: “I don’t think the police will have time to control the black-market sales of tobacco. They don’t even have time to control simple little crimes that happen.”

Others anticipated personal safety would decrease as people who smoke in public became crime targets. Amelia commented: “If someone’s walking down the street with a cigarette, people might take their handbag or whatever.” Ivan foresaw more opportunistic crime as people succumbed to temptation; he surmised: “young people… they’ll see a pack of smokes in a car and they[‘ll] end up breaking into it. You know, it’s just, it’s just, [the proposed retail policy] not going to help anyone.” More generally, participants believed gangs would gain greater control over tobacco supply, leading to a more entrenched black market: “it will cause the gangs to take over everything now…I truly do think that will probably happen” (Rita).

Several thought outlets able to continue selling tobacco products could become targets of concentrated criminal activity; Olivia commented: “So perhaps the risk of it [the store selling tobacco] being targeted is much higher because you’re taking the risk from all of those little places into one place.” Yet, a minority thought designated tobacco outlets would have better security; as Tui noted: “if cigarettes weren’t in the shops, they wouldn’t be ram raiding… it’s a lot bigger thing to ram raid a [expletive] Pak’n’Save [supermarket] than smaller town outlets… having bollards in place obviously does deter that sort of thing.”

### Complex Supply Networks

As tobacco outlets decreased, participants believed supply networks would become more complex, though their views varied. Some expected illicit supply to remain at low levels, others anticipated scalping practices would increase, and yet others believed cheaper tobacco would become available.

A small minority thought illicit tobacco would remain a niche market for those “in the know”; Steve commented: “that market is very like, it’s not easy to get into… once you do have the contact, yep, you get into it, otherwise, no….” However, most thought illicit supply would grow, particularly among desperate people who could be exploited. Pablo explained: “You’ll probably get a lot of schemers… up and coming entrepreneurs” who would charge extortionate prices that people far from designated outlets would be forced to pay. Dora predicted: “People from the suburbs cannot get to those [designated tobacco stores]… it’s gonna cause a black market to grow and someone’s gonna get rich out of it… just ripping people off, but if people are desperate, they’ll pay that money.”

Yet while some participants expected illicit tobacco to be more expensive as “entrepreneurs” charged exorbitant prices, others anticipated lower prices. Fabian suggested prices would probably be “a lot cheaper than… supermarkets” and Uri thought the cost differences between illicit and licit tobacco would be so large that “people … would just rather risk buying them illegally than having to pay… the store prices.” Sophia recalled stockpiling that had occurred during COVID-19 lockdowns and thought some people would accumulate tobacco for on-selling, though she expected prices to fall: “Everybody stocking up on stuff and I feel like that’s probably going to happen… the word will get around, you know, blah blah blah has got some cigarettes… and then I’m gonna come get some cigarettes cheap.”

While the prospect of reduced prices appealed to some participants—Rita commented that she was “always on a hunt for a bargain”—others felt unmotivated to locate illicit tobacco traders and thought smoking could become too problematic to continue. Bryan noted: “I couldn’t be bothered going through [a] black market to buy tobacco; I’d rather just quit and be done with it… say nah, I’m done, I’m over it.”

### Limited Knowledge and Experience

Despite their expectation illicit trade would grow, only a small minority reported purchasing tobacco they knew had been illegally imported or stolen. Steve commented: “somebody had told me that somebody was selling cigarettes [a contraband Chinese brand] for $20 a packet so I started going there for a little bit…then I hear they got busted, so, I wasn’t able to go there.” He explained his purchases: “I was only buying from him for like two months… every second week… really just if I needed to save the money, otherwise… I had no problem spending another $10 on a pack.” Steve’s purchases were opportunistic, infrequent, and short-lived; only one participant (Tui) regularly bought stolen tobacco, though her supply was also sporadic: “my circles include those of us who are living day to day*…* [I’m not] …in any way, shape or form condoning what they do [ram raids on retail outlets], [but] I’m rapt when those young fellas come round [because] that’s actually like saving money.”

When probed, participants thought suppliers would use social media to promote illicit tobacco; Steve noted: “you usually see the articles on Facebook ‘this place was ram raided’… then all of a sudden, they’re somehow off, selling them online… for like 20 bucks.” However, he went on to explain that groups featuring stolen tobacco posts were typically private, to avoid detection. Overall, few participants could explain how illicit markets would become established or how people would gain entry to these sources.

Several participants had tried homegrown tobacco supplied by friends or family members. Almost invariably, they found this tobacco unpleasant and described homegrown tobacco as “disgusting” (Elsie), “feral” (Olivia); “foul” (Rita and Vera), and “rubbish” (Tatiana). Because of its low palatability, using homegrown tobacco was not perceived as a long-term solution but rather as a stopgap measure until they could stop smoking. Atarangi commented: “my dad and I have already discussed it… he’s, like, 40 years a smoker… he lives in [small town] so I couldn’t imagine there’ll be tobacco outlets there… our next option [would be] to grow our own… I don’t enjoy the taste of grow-your-own tobacco; it would lead to me actually giving up.”

More generally, concerns about the quality and safety of illicit tobacco from any source reduced participants’ interest in this market. Whina felt “quite particular when it comes to my cigarettes” while Bryan anticipated “a quality issue…if it’s a black market, there’s no regulation on what strength it is or how it tastes.” These concerns applied particularly to illegal sales of homegrown tobacco, which participants saw as even riskier than legally sourced products. Atarangi explained: “I know we don’t know what’s going into our tobacco right now, but it’s not as bad as what people can mix it with when they grow their own and sell it. It’s quite a scary game.”

### Specific and Societal Solutions

Few participants could identify solutions that would address the growth in illicit trade they foresaw and most suggested rescinding the retail reduction policy and “just leaving people be” (Atarangi). However, others proposed limiting purchases to reduce stockpiling and speculation; implementing the retail reduction policy gradually, so people could establish new (legitimate) supply routes or quit, or offering free nicotine replacement therapy to support cessation.

A minority saw illicit markets as an indication of deeper societal problems and proposed more fundamental changes, including comprehensive responses to people experiencing poverty. Tui argued: “why is this happening? They’ve been driven to it… do you think this [ram raids on retail outlets] would really be happening if they weren’t already starving?.” She presented ram raids as a distributive mechanism and coping strategy: “they’re not trying to attack their parents or their grandmother… it’s not something personal, in their mind… if you’re thieving at a corporate level like that, really, it’s all written off, isn’t it?.”

## Discussion

We identified several gaps between participants’ experiences and expectations. While they anticipated illicit tobacco use would increase if supply of legal tobacco decreased, nearly all differentiated themselves from the people who they thought would use these supply routes. Many predicted public safety would decline once fewer outlets sold tobacco and foresaw a growth in gang power, yet few had knowingly bought illicit tobacco and most knew little about how they could access this market. The only participant with experience of stolen tobacco thought limiting sales to larger more secure stores would reduce “ram raids” and stolen tobacco sources, thus potentially reducing gang power. Participants agreed legal tobacco could be stockpiled; however, some thought stolen tobacco would reduce prices while others expected illicit tobacco to be more expensive, though more accessible, than legal products. Despite finding lower-cost tobacco appealing, several viewed illicit tobacco, particularly homegrown products, as unsafe and unpalatable, and felt unwilling to purchase it. Among those who suggested responses to illicit trade, most saw rescinding the policy and maintaining the status quo as the best solution; only a small minority suggested specific responses or considered how social factors, such as poverty, supported illicit market growth.

The small number of people who reported purchasing smuggled foreign purchases was similar to independent estimates of illicit trade in Aotearoa NZ, which suggest around five percent of packaged products are illegally imported.^28–31^ These estimates are lower than those reported in other countries and reflect, at least in part, NZ’s status as a remote island country with strong border security.^38^

Evidence that illicit product purchases tended to be sporadic, according to availability, contrasts with international studies reporting more established sources of illegally imported tobacco.^39–41^ Qualitative studies from the United States and United Kingdom found participants had high awareness of illicit tobacco, which was widely available in communities experiencing greater deprivation.^39–41^ For example, Stead et al. found illicit trade had become normalized via “fag houses”; although black market tobacco was of lower quality, the purchase process was easy and safe to navigate, and both buyers and sellers benefitted from the arrangement.^40^ By contrast, U.S. illicit tobacco users sourced products from known and established retailers, and preferred to avoid casual “hookups” with street sellers who typically sold lower quality and less palatable tobacco.^39,41^

Although some of our participants thought reduced tobacco availability would mean illicit tobacco commanded extortionate prices, U.S. and UK studies found illicit tobacco was consistently cheaper than legal tobacco.^39–41^ However, these studies discussed illegally imported tobacco and took place in settings where more expensive legal tobacco remained widely available, a difference that may explain how the licit and illicit markets reached a functional equilibrium. Our participants saw illicit tobacco as homegrown or stolen, rather than illegally imported; their ad hoc experiences suggest an unstable market that greater monitoring and increased border security could prevent from reaching equilibrium.

Beliefs that illicit trade would increase did not reflect participants’ past use of illegally sourced commercial tobacco, which few had used. Despite their past experience with homegrown tobacco, few intended to engage with this market, which they saw as a risky way to obtain low-quality products. This discrepancy suggests tobacco companies’ claims about rising illicit trade do not align with how people who smoke intend to respond, once tobacco is less available.^19,22,23,42^ Researchers, advocates and the media could challenge exaggerated predictions that illicit trade growth would follow retail reduction measures, thus preventing this narrative from becoming established.^22^

Although the gaps between perceptions and intended behavior we have identified question industry claims, regulators must continue to prevent illicit markets from developing.^43^ Future research could assess NZ’s approach, which has allowed liberal access to alternative nicotine products, such as vaping devices, and expanded cessation support,^43^ a response suggested by participants in this and other studies.^34,36,44^ Recent provisions that increased funding allocated to border monitoring should provide early warnings of rising illicit imports, but the processes established will require careful evaluation. Furthermore, while its maritime borders create barriers to illicit trade, NZ could nonetheless participate in international networks monitoring tobacco trade. Ratifying the Protocol to Eliminate Illicit Trade in Tobacco Products would support more effective international monitoring of global tobacco supply chain.^45,46^ Should ratification proceed, future research could assess how effectively the intelligence received via this network assisted illicit trade detection.

Future research could also address our study’s limitations; neither interviewer identified as Māori (or Pacific); kaupapa Māori research or Pacific talanoa could have elicited different insights about the role illicit tobacco plays within those communities. While possible that some participants may have withheld details of illicit tobacco use, their apparent openness about their experiences suggests we collected rich and carefully considered data.

Although illicit trade may appear to threaten new policy initiatives, participants’ limited knowledge of these markets and concerns regarding product safety suggest illegal tobacco may pose less of a threat than tobacco companies have claimed. Policy makers should not be deterred by industry arguments. Paradoxically, concerns about illicit tobacco support measures designed to reduce smoking prevalence because, as fewer people smoke, the financial gains illicit suppliers can realize, and thus their interest in developing illegal tobacco markets, will decline.

## Supplementary Material

A Contributorship Form detailing each author’s specific involvement with this content, as well as any supplementary data, are available online at https://academic.oup.com/ntr.

ntad034_suppl_Supplementary_File_1

ntad034_suppl_Supplementary_File_2

ntad034_suppl_Supplementary_File_3

## Data Availability

We have provided additional data from transcripts in a supplementary file but cannot share the interview transcripts because of commitments made to participants that the full transcripts will only be available to people named on the ethics application.
