# Supplementary material for: Perceptions of Illicit Tobacco Sources Following a Proposed Reduction in Tobacco Availability: A Qualitative Analysis of New Zealanders Who Smoke
Source: Nicotine Tob Res. 2023 Mar 4;25(7):1348–54. doi: 10.1093/ntr/ntad034 (PMC10256883; doi:10.1093/ntr/ntad034)
Supplement: ntad034_suppl_Supplementary_File_1 [file ntad034_suppl_supplementary_file_1.pdf]

Reducing Tobacco Retail Outlets

Start of Block: Default Question Block

Reducing Tobacco Retail Outlets: Interviews
Eligibility Questionnaire

To check whether we're able to interview you, we'd like to ask you a few questions about yourself. Please remember that your responses will be kept confidential and you will not be personally identified in any research reports or publications.

1. **How old were you when you had your first puff on a cigarette?**

____

2. **How old were you when you first started to smoke cigarettes <u>at least once a week</u>?**

____

3. **For about how many years have you smoked at least one cigarette each day?**

____

4. **On average, how many cigarettes do you smoke <u>each day</u>?**

____

5. How soon after waking up do you have your first cigarette?

- ◯ Within 5 minutes (1)
- ◯ 6 - 30 minutes (2)
- ◯ 31 -60 minutes (3)
- ◯ After 60 minutes (4)

---

6. What are the <u>main shops</u> where you currently purchase tobacco?
These are shops where you would buy tobacco <u>at least once a week</u>.
Please write the store names, streets, suburbs and city below

Example store: ***Four Square, Forbury Road, St Clair, Dunedin***

- ◯ Main store 1 (4) ____
- ◯ Main shop 2 (5) ____
- ◯ Main shop 3 (6) ____
- ◯ Main shop 4 (7) ____
- ◯ Main shop 5 (8) ____

---

7. Have you ever deliberately tried to quit smoking for at least 24 hours? (i.e., you <u>chose</u> to not smoke for at least 24 hours)

- ◯ Yes, within the last 6 months (1)
- ◯ Yes, more than 6 months ago (2)
- ◯ No, I have never tried to quit smoking for more than 24 hours (3)

---

8. If you wish to be interviewed and are eligible, how will you take part in this interview?

- ○ By phone - audio/voice only (1)
- ○ By phone- audio and video (2)
- ○ Using a tablet (3)
- ○ Using a laptop or PC (4)

---

9. What is your date of birth?
dd/mm/yyyy

____

---

10. Which of these do you <u>most</u> identify with?

- ○ Male (1)
- ○ Female (2)
- ○ Gender diverse (3)

---

11. Which ethnic group(s) do you identify with?
Please tick all that apply

- ☐ New Zealand European (1)
- ☐ Māori (2)
- ☐ Samoan (3)
- ☐ Cook Island Māori (4)
- ☐ Tongan (5)
- ☐ Niuean (6)
- ☐ Chinese (7)
- ☐ Indian (8)
- ☐ Other (such as Dutch, Japanese, Tokelauan) (9)

____

So we can contact you, what is your phone number?

____

What is your email address?

____

What is the best way to get in touch with you?

- ○ Email (4)
- ○ Phone (5)

Thank you for your help with our research

Please click on the arrow to record your answers

End of Block: Default Question Block
