# Supplementary material for: Perceptions of Illicit Tobacco Sources Following a Proposed Reduction in Tobacco Availability: A Qualitative Analysis of New Zealanders Who Smoke
Source: Nicotine Tob Res. 2023 Mar 4;25(7):1348–54. doi: 10.1093/ntr/ntad034 (PMC10256883; doi:10.1093/ntr/ntad034)
Supplement: ntad034_suppl_Supplementary_File_2 [file ntad034_suppl_supplementary_file_2.pdf]

Supplementary File 2

Reducing Tobacco Retail Outlets: Interviews

Interview guide

Introduction
Hello I'm [interviewer name]; I arranged to meet with you to talk about how people who smoke might respond to a reduction in the number of tobacco retail outlets.

Interviewer personal introduction as appropriate for participant (may be modified depending on prior phone discussions)

Removed for interviewer privacy

Would you like me to open our meeting with a karakia?

Proposed karakia to open discussion:
Whakataka te hau ki te uru
Whakataka te hau ki te tonga
Kia mākinakina ki uta
Kia mātaratara ki tai
E hī ake ana te atakura
He tio, he huka, he hau hū
Tīhei mauri ora!

Before we start I'd like to remind you about your rights as a participant in the research project. We have gone through the information sheet with you when we phoned to see if you had any questions about the study. Are there any questions that you'd like us to answer now? Here is a copy of the consent form; please take a few moments to look through it. **Before I go through the consent form, could I please turn on the audio recorder. I am using the record function on Zoom and an online transcribing package called Otter.ai.**

- **SCREEN SHARE CONSENT FORM AND OUTLINE KEY POINTS WITH INTERVIEWEE; ASK IF THEY HAVE QUESTIONS AND ADDRESS THESE.**

Just while the recorder is running, I need to go through some key points with you:

- Your participation in the project is entirely voluntary and you can withdraw from the project at any time up to or during my interview. You have the right to ask questions at any time; if there is something that is not clear, please tell me and I will do my best to answer your questions.
- We will destroy personal identifying information including your contact details and the audio recordings will be destroyed at the conclusion of the project. We will keep the transcripts for at least five years.
- This project involves an open-questioning technique; that means we haven't determined all the questions ahead of time and some of these will depend on how our conversation develops. You always have the right to decide if you would prefer not to answer some questions, so if we come to a topic you don't feel comfortable discussing, please let me know and we will move on.
- You also have the right to stop the interview and withdraw from the study at any time **up until the point the interview ends**. If you decide to withdraw from the study, there is no penalty.
- We will offer you a $40 gift voucher to recognise your time and assistance. The project is funded by the New Zealand Cancer Society and there will be no commercial use of the data obtained.
- We aim to publish the results of the project and present these at meetings; the University library will have a copy of any published work we prepare and we will send a copy of your interview transcript and interview summary. We will also send you a summary of the overall project findings. We will use our best efforts to keep your responses confidential to members of the research team named on this sheet.

- **DO YOU HAVE ANY QUESTIONS BEFORE WE GET STARTED?**
  PLEASE REMEMBER THAT YOU CAN ASK ME QUESTIONS AT ANY TIME DURING THE SESSION.

- **CAN YOU PLEASE CONFIRM THAT YOU CONSENT TO TAKE PART IN THE INTERVIEW? (ENSURE CONSENT IS RECORDED) IF YOU ARE WILLING TO PARTICIPATE, PLEASE SAY "I CONSENT TO TAKE PART IN THE INTERVIEW".**

We are offering everyone a $40 gift voucher to thank them for contributing to our work. Could you please let me know if you would like a voucher from: Farmer's or Bunnings

Personal smoking

1. I'd like to start by asking about when you first started smoking. You indicated that you were __ years old when you had your first cigarette? What was it like? Where did you get the cigarette from? Who were you with? Where were you?

2. You said you were __ years old when you first started smoking at least weekly. When did you start smoking every day? What was it about smoking that led you to smoke more often? Do you smoke RYO (roll your own) or TM (tailor made)?

3. Thinking about your smoking now, you said you smoke around __ cigarettes each day. Does your smoking ever go up or down? When do you find it goes up? And down?

4. You said you had [insert details of quitting ____.

   What prompted you to try and quit? How did you go about trying to quit? What sort of things do you think would make it easier for you to quit?

5. What do you hope will happen with your smoking in the next few years?

Sourcing tobacco – current situation

6. I'd now like to ask you some questions about where you get your tobacco from. About how often do you buy a pack/pouch?

7. I'm going to share my screen with you so that I can show you a Google map of your town.
   [To start, all lists should be ticked OFF in the map legend. Only the base map should be visible]

   Please look at this map on your screen. To begin, I'm just going to show you around for a moment, to help you get your bearings. Here we can see the peninsula; moving left we can see the harbour coming in towards South Dunedin and the downtown; this area here is South Dunedin; the downtown area is up here, including the Octagon right here; and North Dunedin is up in this area.

   Now, I'm just going to make a slight adjustment to the map.
   [Tick ON the list labelled 'Current Retailers-all City' in the map legend]]

   You'll see now that we've used red pins to mark most of the retailers that currently sell tobacco in Dunedin. I'll give you a minute or so to take a look. If you have any questions about what you're seeing, please let me know.
   [Pause, allowing participant to look. Probe further to help with orientation]

   - To help you get your bearings, would you like me to point out any more key locations around the city as points of reference on the map?"
   - Would you like more time to look at the map? **[Pause to review map further, if requested]**

   Now, I'm going to highlight ONLY those places where you normally buy your cigarettes or tobacco. These are the stores you told us about in the survey you filled out for this study.
   [Tick OFF the list labelled 'Current Retailers-all City' in the map legend. Tick ON the list labelled 'Current retailers- interviewee']

   Let's just go through these one by one. Looking at this store **[name first store from on-boarding survey, hovering over it with curser and letting participant know that you are doing this]:**
   What are the main reasons you get your tobacco from this store? [**If necessary, probe further re. how that particular outlet fits into their routine, and the time it takes to reach the outlet from wherever any reference point(s) might be that they've specifically mentioned]**

   What about ___? [**repeat until all stores mentioned have been explored]**

[STOP screen sharing with interviewee for the next two questions; map will be re-introduced in the following section]

8. Aside from buying from shops, are there any other places you get cigarettes from? **Pause for participant to respond. Follow up by probing all options not mentioned.**
Have you ever bought tobacco… online? Have you ever tried to grow your own tobacco? Have you ever bought tobacco from someone selling on the street or from a private home? What about from a market or other community venue?

9. What makes you go to those sources/places for tobacco? Do you buy the tobacco, or is it given to you? How easy is it to get tobacco from [relevant non-retail source]? How often do you go to xxx? What about xxx…?How much does it cost compared to buying from your regular shop(s)?

10. Do you know of any other ways of getting tobacco? Can you tell me about those? Have you ever used those sources? What made you try that source?

Sourcing tobacco – future situation

[Google map to be shown again via screensharing. Tick ON lists labelled 'Current Retailers-all city' and "Current retailers- interviewee"]

I'm now going to share my screen with you once again.

Here, you can see the same Google map of your town that we looked at before. We've used red pins to mark most of the retailers that currently sell tobacco in Dunedin. We've also used blue pins to highlight the places where you normally buy your cigarettes or tobacco, with store names written alongside them. I'll give you a moment to take a look. If you have any questions about what you're seeing, just let me know.

11. I'm now going to ask you what you think about a government policy that will greatly reduce the number of tobacco retail shops in your town and across the country. The details of this policy plan haven't yet been released, but the number of tobacco outlets throughout the country could drop from around 8000 to around 500. It's likely that very few dairies and petrol stations will still be able to sell tobacco.

So, at the moment you can buy tobacco from the stores you've indicated. Once the new policy is introduced, however, only a small number of stores will sell tobacco.

I'm now going to show you an example of what this policy change might look like in Dunedin.

[Tick OFF lists labelled "Current retailers- all city" and "Current Retailers- interviewee". Tick ON list labelled "Retailers post-changes"]

Now we've tried to show what the new policy might look like. This time we've used green pins to show the possible number of stores you'll be able to buy tobacco from in Dunedin after the policy comes into effect.

[CHECK: Does participant want to see before and after again? Would they like to see current retailers alongside the proposed changes? Do they have any further questions about what they are seeing?]

12. What do you think of this change in the number of shops selling tobacco? What do you think about the restricting the type of shops allowed to sell tobacco to just NN in your area **[personalise for each participant depending on location]**?
13. If tobacco is taken out of dairies and service stations, what sort of stores do you think should sell tobacco? What makes you think these would be suitable stores for selling tobacco?
14. If only a few stores sell tobacco in your town, how would you feel about that? How would you adjust to the changes? **Probe:** Change where you buy tobacco? Look for people selling tobacco on the street? Grow your own? What other things would you consider doing? How easy or difficult would it be to make those changes?
15. How do you think having fewer stores selling tobacco would affect the amount of tobacco you normally buy at a time? **Probe:** do you think you might purchase less, purchase more, plan/save for purchases, group buy, bulk buy? How would these changes affect your financial situation? How do you think you would handle that change?
16. Would having fewer stores selling tobacco affect how often you smoke? **Probe:** Would you smoke more, less, or about the same? **If change noted, ask:** how would that affect your daily smoking pattern? What effect would these changes have on your experience of smoking ?
17. To what extent would having fewer places selling tobacco get you thinking about quitting? Or trying to switch to vaping?

Now, I'm going to ask you a series of questions that relate to <u>other</u> people's smoking behaviour. Please disregard the map for a few moments while I make a couple of changes.

[In the map legend, tick OFF the list labelled 'Retailers post-changes'. Tick ON list labelled 'Current Retailers-all City']

Please look at the map on your screen. Once again, you're looking at most of the retailers who currently sell tobacco in Dunedin. These are indicated with red pins.

[Tick OFF the list labelled 'Current Retailers-all City'. Tick ON the list labelled 'Retailers post-changes']

And once again, here is an example of where people could buy tobacco in Dunedin once the new policy comes into effect. Again, we've used green pins to show the possible number of stores selling tobacco after the policy is introduced. **[CHECK: Does participant want to see before and after again? Would they like to see current retailers alongside the proposed changes? Do they have any further questions about what they are seeing?]**

18. How do you think having fewer stores selling tobacco might affect other people who smoke? [purchase locations, purchase quantity and financial impact, amount smoked, quit attempts…]
19. How do you think the changes might affect people who are thinking about quitting? What about people who have recently stopped smoking within the last few weeks or months? Or people who stopped smoking a year or more ago? How do you think these changes might affect people's willingness to try vaping instead of smoking?
20. How do you think having fewer stores selling tobacco might affect young people who want to try smoking? What about young people who have already started smoking? How do you think these changes might affect young people and their willingness to vape [check for impact on never smokers and current smokers]?
21. How do you think having fewer stores selling tobacco might affect the way other people think about people who smoke? **Probe: Mmm or how do you feel about that?** What effect would having fewer

stores selling tobacco your perceptions of smoking? Are there any other ways you think you might be affected?

22. Do you think having fewer stores selling tobacco could lead to any unintended outcomes? What do you think these would be? How would these affect you? What would you do to respond? What do you think other people who smoke would do?
23. Some groups have commented on these changes. A group representing some dairy owners has said that tobacco sales are essential to their businesses and that if they couldn't sell tobacco, they would go out of business. What do you think of this argument? What do you think could be done to help dairies and small businesses transition away from selling tobacco?
24. Some research from Aotearoa New Zealand has found that tobacco and cigarettes make up only a small percentage of the purchases people make at dairies and the researchers have questioned dairy owners' concerns. Does this research affect how you see dairy owners' arguments? In what way?
25. The dairy owners' group also claim that restricting the sale of tobacco will lead to a bigger black market controlled by gangs. What do you think of this argument? How big a problem do you think black market tobacco will be? How could black market tobacco be better controlled?
26. Some people think it's important that the Government introduce policies to improve people's health but other people think the Government should stay out of people's lives and some people don't really care. What are your thoughts?
27. Are there any benefits to these changes that we haven't talked about yet? Can you tell me about these?
28. Are there any negatives we haven't talked about yet? Can you tell me about these?

Summary

29. These are all the questions I wanted to ask you. Do you have any other comments you'd like to make? Is there anything we haven't discussed that you'd like to comment on?

IF IN PERSON, ENSURE PARTICIPANTS SIGN TO ACKNOWLEDGE RECEIPT OF GIFT VOUCHER; ENSURE VOUCHER NUMBER IS RECORDED

IF MEETING BEGAN WITH KARAKIA PLEASE NOTE:
Thank you again for making time to share your ideas with us. I'd like to close our meeting with a karakia:
Unuhia, unuhia
Unuhia ki te uru tapu nui
Kia wātea, kia māmā, te ngākau, te tinana, te wairua i te ara takatā
Koia rā e Rongo, whakairia ake ki runga
Kia tina! TINA! Hui e! TĀIKI E!
