# Supplementary material for: Perceptions of Illicit Tobacco Sources Following a Proposed Reduction in Tobacco Availability: A Qualitative Analysis of New Zealanders Who Smoke
Source: Nicotine Tob Res. 2023 Mar 4;25(7):1348–54. doi: 10.1093/ntr/ntad034 (PMC10256883; doi:10.1093/ntr/ntad034)
Supplement: ntad034_suppl_Supplementary_File_3 [file ntad034_suppl_supplementary_file_3.pdf]

Supplementary File 3: Summary Code Book	
**Theme 1**	**Key quotations to illustrate theme**
Increasing marginalisation and crime	**Expectations of illicit trade and crime**<br>Atarangi<br>I think the crime rate is going to shoot through the roof. It's really bad. You know, since the cigarette prices have gone up slowly over the last few years, all you see ram raids. We see as robberies, they want that tobacco, they want to sell it they want to smoke it because they can't afford it. I think that if this gets made public, I'm, gonna feel sorry for dairy owners and gas stations, because people are gonna steal as much as they can. Because it's not gonna be available for them.<br><br>Dora<br>What I would worry about is people attacking people in supermarkets for cigarettes.<br><br>Elsie:<br>Probably steal. I'd say steal money or probably use harm to get it out to people maybe... if you're desperate enough... if you're lonely enough and desperate enough... cuz I've been in that place... however you bloody well can. So, there needs to be proper decisions around that as well because, ummm, you know, if they want to really get rid of tobacco altogether, I just think that there's just gonna be like... I think this is gonna cause a lot of trouble.<br><br>Harriet<br>Probably just like the full time smokers, the heavy smokers. The ones who have always got to have like, cigarettes or the ones who can't really afford cigarettes, but they'll just go and help themselves and cause harm to other people.<br><br>**Growing gang involvement**<br>Jake<br>gangs will probably …there will would most likely be a black market on tobacco. And it would most likely be run by the gangs to make money.<br><br>Leanne<br>once you've got a black market that needs to be funded and it can't be funded from legitimate money, which means crime increases to fund it.... You are giving people a choice between breaking the law, and feeding their addiction. And the group that we're concerned about will feed their addiction so you're teaching them to break the law. So you move a whole sector of society, who currently is simply an addict, into becoming a criminal addict. And I'm not sure how many of those that then get sucked into all of the other things that go on in the black market community where people openly make money out of breaking the law because once you've stepped across into one criminal activity, there's nothing to stop you.

	Nicolas<br>And you have a couple of people come in [to a store selling tobacco] at any given time of the day, holding up the place, to, get the cigarettes. You're gonna have a lot more people in danger. You know, it's going to be, it's going to be, you know, not an easier target, but it would just be a bit more worrying.<br><br>Rita<br>Of course, it will cause the gangs… to take over everything now. Yeah, I truly do think that will probably happen.<br><br>**Increased risk to stores**<br>Nicolas<br>And you have a couple of people come in at any given time of the day, holding up the place, to, get the cigarettes. You're gonna have a lot more people in danger. You know, it's going to be, it's going to be, you know, not an easier target, but it would just be a bit more worrying..<br><br>Olivia<br>I think because you know, places who sell them, are targeted for that product, then that's going to all combine into those three locations. So perhaps the risk of it being targeted is much higher, because you're, you're taking the risk from all of those little places into one place. As this the only availability.<br><br>Rita<br>Because how many security guards can you put at a supermarket? It's pretty incredible if people want it, they go, they think they're entitled to it, so they just go for it. I don't understand some people's logic<br><br>Tatiana<br>It's already happening in our supermarkets. Hamilton, I'm not too sure if you're clued up with our pages, but, people just walk in, do their shopping, they walk out because they can. Nobody can stop them. So what's to say that it's not going to happen with tobacco as well?<br><br>**Decreased risk to larger stores**<br>Amelia<br>I think much more better they would have you know, like as it is at the moment, you know, things are all locked up. They don't advertise it anymore. Things like that. And yeah, they've got some good systems in place already and fact they've got a lot of staff there.
Theme 2	**Exploitation of vulnerable people**

Complex supply networks	Dora<br>Because people from the suburbs cannot get to those... yeah, it's gonna cause a black market to grow and someone's gonna get rich out of it. ….Well someone [inaudible] ... got money would go in and buy, say, 20 cartons, take them out to the suburbs and sell them at double the value. Just ripping people off but if people are desperate they'll pay that money.<br><br>Harriet<br>It's probably just like, the people who are selling the cigarettes to try and get as many cigarettes, that's my own opinion. ... trying to get like as many cigarettes and then they'll take them and then, of course, put them on the black market and then, raise the price on them and it'd cause a bit of a strife<br><br>Pablo: You'll probably get a lot of schemers. You'll probably get a whole lot of up and coming entrepreneurs…. I mean the little you know, entrepreneurs people…. Get it online. You know all that bunch that smoke. And start ... getting it to family members or whatever.<br><br>Steve:<br>Just the fact that ... just the convenience like now, you can't go anywhere for them and maybe somebody's going to be in an area where they've kind of got this market for people that want to buy cigarettes but can't go out and buy them so they're gonna get somebody to, steal them and then kind of on sell them and then make a profit off that that's what I see happening, which is already happening already. But, I wouldn't be surprised if it did increase.<br><br>Steve:<br>I think that maybe when this policy comes through, I think it's gonna give people the kind of money, the money, money signs in their eyes and be like, okay, this could be our way to kind of push through a product and supply them and make some money. I think people are just going to look at it as a money opportunity.<br><br>**Lower cost product will be appealing:**<br>Fabian<br>Oh I think it could "...". I think it would happen a lot more. They probably sell it a lot cheaper than what….supermarkets or whoever<br><br>Nicolas<br>You know, put it this way. If, if, if I can get it cheaper from somewhere else, legal or not, I'll probably do it.<br><br>Uri

there already is a black market for it, because it's expensive, ridiculously so. So that black market already exists just simply out of the fact that buying cigarettes is such a dent, in the someone's wallet.

Wiremu
If it's there and it's offered and it's cheaper, then yeah of course

Quality and safety concerns
Bryan:
…there's a very high possibility that it may become a black market thing but then... you've got a quality issue. As what, like you what you get now is a controlled quantity of what it is, like, if it's a black market there's no, no regulation on what, what strength it is or how it tastes

Jake
Just don't know what people do to them.

Olivia
No. I will not trust anyone off the street. You ?? know, you just never know. Like really don't. Anybody would do anything to make a quick buck. You know, so I just ah, I know how that scene operates and I just wouldn't [go there]

Whina
I'd definitely be questioning that and that's what I mean by people don't approach me because I question it. Like why are those ten dollars, like where did you get those from? You know, but in saying that ... I know a whole lot of people that would. Yep, I would say probably most of the people I know that smoke would, would yeah, of course we'll buy ten dollar cigarettes

Home grown tobacco quality concerns
Olivia
I've had homegrown before but it's feral.

Rita
Oh no not me, but I've tried to smoke homegrown and it's foul. It doesn't matter how much you mix it with another packet. You can still taste that.

Tatiana
I thought it was rubbish. It was too harsh.

	Vera<br>I have bought from somebody else that has grown their own. But it's bloody foul. (laughing)<br><br>Whina<br>maybe about 15 years ago, maybe, would have been about 20. Maybe a little bit younger than 20. My cousin had some, here, in ... in Raglan actually. She had some like homegrown. ... But I think she had actually tried to grow that herself. And it was, it was terrible. Absolutely terrible ??. Yeah. Now that I looking back here. My cousin had some.

**Theme 3**<br><br>**Limited experience and knowledge of illicit tobacco**	**Past experience of illicit markets**<br>Steve:<br>Oh, it was pretty much just somebody had told me that somebody was selling cigarettes for $20 a packet. So I started going there for a little bit when I was living in Rotorua and then I hear they got busted, so, I wasn't able to go there so I had to buy it legally again.<br><br>Tatiana<br>I have purchased and I don't like it…. It was just a one off. I'd never purchase off anybody. Except for a store. Yeah.<br><br>**Accessing illicit markets**<br>Rita:<br>Yeah. Yeah. It can be a mixture of both they don't, like, you know, you can buy marijuana on Facebook. It's not hard to get. It's, it's going to be exactly the same for tobacco.<br><br>Sophia<br>Word of mouth, or you know, who knows where to get some cheap cigarettes? Is anyone selling in? And I that's what I'm thinking. No. That some, you know, some people will probably try to do that. Load up on cigarettes, and then, you know, sell them privately. Or, you know, let's get the word out. They'll get the word out, and then I'm gonna come get some cigarettes cheap. ?? cigarettes. I think other people would try to do that. Sell them privately.<br><br>**Limited interest**<br>Atarangi:<br>If you asked me this year ago would have said yes. But because of my, my career prospects and what I want to do with my future I cannot afford any legal activity. So I, don't think I would go down that road. ... It'll just I mean, getting, not being able to become a [career path] because I went and bought a packet illegally. That's not worth it to me.<br><br>Ivan<br>Well, I'm not 100 % sure [how big a problem BM products will become], cos I don't really deal with any of that sort of stuff, but ... Like I'd I'd imagine I imagine it was slowly seep into becoming a, like a bigger problem…. Probably not [a big problem] because not a lot of people really look for that sort of stuff.<br><br>Leanne<br>Because the fact that I'm an addict doesn't actually put me into the seedy side of doing deals on street corners. Also, part of that is because I've accepted the price I pay through a legitimate business. I'm not trying to avoid yeah, I, I just, I am not the kind of person

	to actually associate with people hocking things off on street corners for a start off. I don't imagine they would want to me anywhere near them. … People like me don't buy off street corners.<br><br>it's a little bit difficult. Because I really don't, I really don't understand how it works because I really don't use the black market at all.

Theme 4<br><br>***Specific and society solutions***	**Limiting purchases**<br>Dora:<br>When they have only [some] outlets, not to let people bulk buy. Not to let people bulk buy. Okay. Put a limit on it. You can get the maximum of three packets.<br><br>**NRT**<br>Amelia:<br>I guess offering free nicotine therapy for all.... Ahhh. I think that would help some people that are on low incomes and limited cigarettes and stuff.<br><br>**Gradual implementation:**<br>Vera:<br>By not taking away all of it at one time. Like to gradually do it.<br><br>**Retaining status quo**<br>Amelia<br>Put cigarettes back in the dairy<br><br>Ivan<br>Well yeah, the only thing I could really, say would be to, basically, you know go against this policy.
